# Parenting with nutrition education and unconditional cash reduce maternal depressive symptoms and improve quality of life: findings from a cluster randomised controlled trial in urban Bangladesh

**DOI:** 10.1080/16549716.2024.2426784

**Published:** 2024-11-19

**Authors:** Sheikh Jamal Hossain, Fahmida Tofail, Anisur Rahman, Jane Fisher, Jena Derakhshani Hamadani, Syed Moshfiqur Rahman

**Affiliations:** a Global Health and Migration Unit, Department of Women’s and Children’s Health, Uppsala University, Uppsala, Sweden; b Maternal and Child Health Division (MCHD), International Centre for Diarrhoeal Disease Research Bangladesh (icddr, b), Mohakhali, Dhaka, Bangladesh; c Nutrition Research Division (NRD), International Centre for Diarrhoeal Disease Research Bangladesh (icddr,b), Mohakhali, Dhaka, Bangladesh; d Global and Women’s Health, School of Public Health and Preventive Medicine, Monash University, Melbourne, VIC, Australia

**Keywords:** Parenting, unconditional cash, maternal depression, child development, quality of life

## Abstract

**Background:**

Maternal post-natal depression is a global public health problem. Parenting interventions targeting children’s development may also bring benefits to mothers, but few parenting interventions have been studied thoroughly.

**Objectives:**

The study aimed to measure the effect of a parenting intervention using culturally appropriate and locally made toys, along with nutrition education and unconditional cash, on maternal depressive symptoms (MDS) and quality of life (QoL).

**Methods:**

The study was a cluster randomised controlled trial with two arms: i) intervention: parenting with nutrition education and unconditional cash and ii) comparison: unconditional cash in an urban setting in Bangladesh. Twenty clusters were randomised to either the intervention or control group. Community Health Workers (CHWs) delivered parenting and nutrition education sessions fortnightly in households for one year. The participants were mother-child (6–16 months) dyads. The MDS and QoL were measured using the Self-Reporting Questionnaire-20 and a brief version of the QoL questionnaire. Linear regression analysis was used to assess the treatment effects.

**Results:**

After one year of intervention, 547 mothers (93%) completed the study. The mothers in the intervention group had lower MDS [Regression coefficient (β)=-1.53, Confidence interval (95% CI)=-2.28, −0.80] and higher QoL scores in physical health [β = 4.21 (95% CI = 1.71, 6.73)], psychological health [β = 3.14 (95% CI = 1.10, 5.19)], social relationships [β = 3.21 (95% CI = 0.76, 5.66)] and environment [β = 3.40 (95% CI = 1.37, 5.44)] compared with the comparison group.

**Conclusion:**

Parenting interventions including nutrition education and unconditional cash, aimed at improving children’s development, resulted in a reduction in maternal depressive symptoms and improvement in quality of life.

## Paper CONTEXT

- **Main findings:** A package of parenting and nutrition education, delivered through a national social safety-net programme (unconditional cash transfer programme), significantly reduced maternal depressive symptoms and improved all domains of quality of life for disadvantaged 40 mothers in urban Bangladesh.
- **Added knowledge:** This study contributes evidence regarding the reduction of maternal depressive symptoms and the improvement of quality of life for impoverished individuals living in urban areas of Bangladesh as a by-product of the intervention.
- **Global health impact on policy and action:** The findings of this study help inform public health policymakers who aim to reduce maternal depressive symptoms and improve quality of life in low-resource settings, such as Bangladesh.

## Background

Maternal post-natal depression is a global problem and is very common in low- and middle-income countries (LMICs) [1]. It affects 10–15% of women in high-income countries [2], whereas its prevalence is estimated between 5–60% in LMICs [3]. Women who have experienced post-natal depressive symptoms have a 30% to 40% likelihood of symptom recurrence or persistence for years unless they receive treatment [4]. Several factors increase maternal depressive symptoms (MDS) [5], including violence against mothers [6,7]. Mothers’ experiences of violence victimisation are negatively associated with their capacity to parent young children [8]. Untreated MDS diminishes mothers’ ability to provide sensitive and responsive parenting. Numerous studies have shown that MDS is a determinant of a range of adverse child developmental outcomes, including delayed cognitive and physical development, an increase in behavioural problems, and subsequent common mental disorders [9–11]. Similar to maternal mental health [12,13], maternal QoL is also crucial for child development [14]. Indeed, children’s linear growth failure and suboptimal early child development (ECD) remain critical problems in LMICs [15,16], which can significantly affect later adult life [16,17].

Findings regarding the association between parenting and MDS are mixed. Some studies indicate that parenting interventions may reduce MDS, while others do not. Evidence-based child developmental interventions, which focus on parenting to improve child development, have also been found effective in reducing MDS in LMICs [18–23]. A systematic review and meta-analysis of nine randomised controlled trials found that parenting intervention can reduce maternal mental health problems [24]. This may be due to that most mothers involved were suffering from depressive symptoms as they were parenting children with symptoms of attention deficit hyperactivity disorder. The findings are further supported by another meta-analysis of 15 studies on parenting interventions conducted in high-income countries, which also showed a significant reduction in MDS [25]. Thus, parenting interventions aimed at improving child development may also have the potential to reduce MDS. Interventions designed specifically for mothers and children between two weeks and six months postpartum have been found effective in reducing MDS [25]. Support during this period is crucial for mothers owing to various biological and psychological factors. However, it remains unclear to what extent and in which contexts parenting interventions are effective in reducing MDS as a treatment outcome [26,27]. Parenting interventions may benefit MDS by fostering maternal behavioural activation, encouraging mothers to engage in pleasurable activities with their children. Such interactions may empower mothers, enabling them to be there for their children, enjoy the process of child-rearing and recognise the benefits of parenting, whilst also feeling valued as members of the family. Conversely, evidence suggests that parenting interventions focused on developmental delay may not be effective in reducing MDS [28,29]. A recent meta-analysis seems to support the notion that parenting programmes may not be effective in reducing MDS [30–32], although some important randomised controlled trials have demonstrated positive effects in terms of reduced MDS [20,21,23]. Factors such as a short intervention duration, lack of quality, reliance on message-based interventions alone, or the presence of severe maternal depressive symptoms requiring clinical treatment may account for the limited impact of some parenting interventions on MDS. Despite findings from earlier reviews supporting parenting interventions for child outcomes in the general population, it is still unclear whether such interventions have a significant effect on MDS during the perinatal period. Specifically, the extent to which these interventions have a positive impact and benefit maternal mental health and QoL is less studied. This article contributes to the limited literature on whether such interventions can reduce maternal MDS and improve QoL in urban settings of LMICs, such as Bangladesh, by using the social protection system through an unconditional cash transfer programme.

Parenting and nutrition education have been found effective in improving children’s cognitive, language and motor development [33]. Moreover, the intervention can reduce household violence against mothers and improve fathers’ engagement, mothers’ knowledge of childcare and the quality of children’s stimulation environments in urban Bangladesh [33]. The urban setting of Bangladesh was selected for this study since there is a lack of research in these areas within LMICs. Urban populations and infrastructures, e.g. health, education facilities, etc., differ significantly from those in rural areas in many LMICs. Thus, it highlights the need for evidence on the effectiveness of such interventions in urban settings.

In this paper, we aimed to evaluate whether the intervention could reduce MDS and improve maternal QoL in an urban setting in Bangladesh. We hypothesised that both the parenting-focused ECD intervention, delivered through fortnightly home visits by community health workers (CHWs), and the unconditional cash transfer would reduce MDS and improve maternal QoL compared with mothers who received only the unconditional cash transfer.

## Methods

### Study design, setting and participants

A cluster-randomised controlled trial was conducted in Rangpur City Corporation, Bangladesh. It was established in 2012, and currently covers an area of 205.70 square kilometres. Higher prevalence of poverty drove us to choose this City Corporation. The participants were enrolled between December 2019 and March 2020.

The study included mothers with children aged 6–16 months who were selected for the urban lactating allowance programme in Bangladesh. These mothers were enrolled in the social safety-net programme of the Ministry of Women and Children’s Affairs, Government of Bangladesh (GoB). The mothers received 800 BDT (9.4 USD, Ref: World Bank 2020) each month as unconditional cash (lactating allowance) during their pregnancy and lactation. The eligibility criteria for being selected as a beneficiary of the lactating allowance were: (i) being an urban working mother (mothers rearing children full-time were also considered working mothers), (ii) income less than 1500 BDT (<17.63 USD) per month per mother, (iii) having fewer than three children, and (iv) permanent residence in the urban area. Further details regarding the participants have been reported elsewhere [34].

### Randomisation and intervention

There were two arms of this cluster randomised controlled trial: i) Intervention: parenting with nutrition education plus unconditional cash and ii) Comparison: unconditional cash only. A *ward*, the administrative unit of the city corporation, served as a clustering unit for the trial. Twenty clusters were randomly allocated to either the intervention or comparison group. In each cluster, thirty mother and child (6–16 months) dyads were enrolled. We collected the list of eligible mothers for the unconditional cash (lactation allowance) programme from the office of the Deputy Director (Rangpur), Department of Women’s Affairs, GoB. We recruited the first thirty relatively younger mothers from the list, if more than thirty eligible participants were available in a cluster. A non-study statistician, who was not involved with the study, randomised the 20 clusters to one of the arms.

CHWs from the *Wards* of the city corporation, who were permanent residents with at least 12 years of education, were recruited for this study. These CHWs received ten days of training on the curriculum for an integrated parenting and nutrition education intervention. The curriculum was adapted from the Reach Up and Play model [35]. The trained CHWs delivered the intervention in the home every fortnight for one year. They demonstrated age adjusted activities for the child by involving both the mother and child in play. Fathers and other caregivers, such as grandmothers or aunts, were encouraged to participate in the sessions if they were at home when the CHW visited. During each session, mothers or caregivers were taught to: i) show consistent love and provide positive feedback for the child’s achievements, ii) be responsive to the child’s behaviour, iii) modify the environment for child and iv) engage in play using toys. The CHWs used culturally appropriate, locally made play materials, such as picture books, puzzles, colourful blocks and plastic bottles for play. Some play materials were left in the home so that the mothers could play and interact with the child as instructed, and these play materials were replaced with others fortnightly during the next session. The CHWs also discussed various topics with the mothers, including nutrition, health and child development. The mothers in the comparison arm received the same amount of cash. The GoB conducted health education sessions separately for all the mothers, but it was not mandatory.

### Sample size

**Figure 1.**
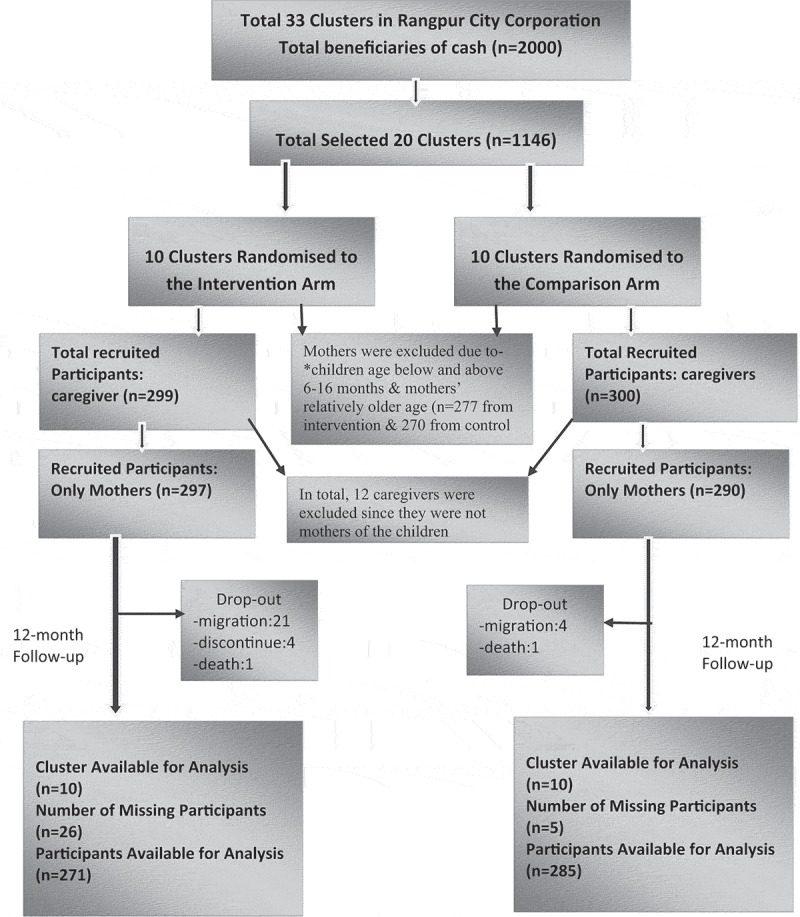
Enrolment and follow-up of the participants in the trial for maternal mental health and quality of life.

The original sample size was 599 mother/caregiver-child dyads, based on achieving a 0.37 standard deviation (SD) improvement in child cognitive development, the main outcome of the study [36]. However, for this analysis, we included 587 mother-child dyads at enrolment, as 12 primary caregivers were individuals other than the mother (e.g. grandmother or aunt) and after the intervention it was 547 (Figure 1).

### Measurement tools

The main outcome of this analysis was MDS, measured using the WHO Self-reporting Questionnaire-20 (SRQ-20), a tool endorsed by the World Health Organization (WHO) [37]. It has been validated in Bangla [38] and other LIMCs [39,40]. The SRQ-20 is simple to use, with a few questions [41]. It measures various symptoms of depression, including headache, poor appetite, sleep disturbances, depressed mood, unhappiness, helplessness and psychomotor retardation. Each question is scored from 0 to 1, depending on the ‘no’ or ‘yes’ response, respectively. The scores are added to generate an overall SRQ-20 score. Higher scores indicate more depressive symptoms [42]. We have previously used this tool in another study [28].

QoL was measured using the quality-of-life questionnaire (bref version) developed by the WHO, known as WHOQOL-BREF. There were 24 items linked to four domains: physical health, psychological health, social relationships, and environment [43]. Thus, the tool can capture multidimensional aspects of QoL. This tool has previously been validated in Bangladesh [44]. All items are rated on a 5-point Likert scale. The domain scores are calculated by multiplying the mean of all facet scores included in each domain by a factor of 4, resulting in a possible range of raw score of 4 to 20 for each domain. These raw scores are then standardised to a scale ranging from 0 to 100, with higher scores indicating a better QoL.

The last experience of household violence during the previous month was assessed using an abbreviated module of the WHO Multi-Country Survey on Violence Against Women questionnaire [45]. The questions were asked in private by female interviewers to ensure confidentiality. Data on the quality of home environmental stimulation were collected using the culturally validated Family Care Indicator tool [46]. Children’s cognitive and language development were measured using the Bayley Scales of Infant and Toddler Development, Third edition [47]. Relevant demographic and socio-economic data were also collected.

### Data collection

Six graduate assessors were trained on all assessment tools for one month. Efforts were made to keep the assessors unaware of the intervention/control status of the mothers during data collection, although mothers may have mentioned participating in some child development sessions. The internal reliability (Cronbach’s Alpha) for the SRQ-20 and WHO-BREF QoL was 0.85 and 0.89, respectively. For each tool, the interobserver agreement was over 90%.

### Data management and analysis

Data were analysed using Stata version 12.1. We segregated the mother’s, father’s and other adults’ involvement scores in child development activities from the Family Care Indicator (FCI) as sub-scores. The summarised baseline characteristics were presented as means and standard deviations (SD) or percentage to assess the imbalance between the groups [48]. Depressive symptom scores were considered to be continuous variables and were normally distributed [49]. QoL scores were calculated following the WHO’s guidelines for QoL-BREF scores [43] and also showed normal distribution.

Multiple linear regression models were conducted for both MDS and QoL (physical health, psychological health, social relationships and environment). The models considered covariates having biological and scientific plausibility with the main outcomes. Household violence against the mother and maternal child developmental activities differed significantly between the groups and were therefore included as adjustments in the model. Clusters were adjusted in each model as a random variable. Respective baseline outcomes and assessor effects were also adjusted in each model. Additionally, to address missing data, multiple imputation by chained equations (MICE) was employed, generating 10 imputed datasets. Rubin’s rules were used to combine the estimates to obtain unbiased findings. The effect size was reported using Cohen’s d, calculated as the difference in the mean change in scores for each domain over the intervention period, divided by the SD of score differences [50].

## Results

At the end of the intervention, a total of 547 mothers were interviewed, and the dropout rate was 6.81% (Figure). In this study population, 54.7% of mothers were suffering from MDS (SRQ score ≥ 7). There were no significant background differences between the intervention and comparison groups, except that mothers in the intervention group participated more frequently in child developmental activities and reported higher instances of household violence (Table I). More detailed group information has been reported in the Lancet Regional Health-Southeast Asia [33].

**Table I.** Baseline characteristics at enrolment by groups (*n* = 587).

Characteristics/ Outcomes	Intervention Mean (SD)/% *n* = 297	Comparison Mean (SD)/% *n* = 290
Mothers’ Age (years)	25.61 (4.77)	25.62 (5.13)
Mother/family paying instalment of loan to microcredit organisation	231 (77.70%)	207 (71.3%)
Mothers’ body mass index (Wt/Ht^2^)	22.75 (3.92)	23.22 (4.51)
Mother exposed to domestic violence	156 (52.52%)	127 (43.79%)
Mothers’ education (years)	7.27 (3.65)	6.96 (3.38)
Fathers’ education (years)	5.71 (4.39)	5.57 (4.17)
Home environment score (Total Family Care Indicators score)	5.78 (3.14)	5.53 (3.30)
Child developmental activities by mother (range 0–5)	2.19 (1.43)	1.94 (1.32)
Child developmental activities by father and other family members (range 0–5)	0.97 (1.57)	0.92(1.67)
Child age (months)	11.46 (3.12)	11.28 (3.24)
Child sex (girls)	142 (47.81%)	152 (51.18%)
**Outcomes**		
Total depressive symptoms score	8.91 (4.87)	8.43(4.65)
Depressive symptoms presence- Yes No	165 (28.1%) 132 (22.5%)	156(26.6%) 134(22.8%)
Quality of life		
Physical health score	52.01 (12.42)	53.09(12.01)
Psychological health score	61.02 (12.01)	60.74 (12.67)
Social relationship score	58.57 (14.73)	58.93 (14.05)
Environment score	50.68 (10.69)	49.48 (11.52)

Values are mean (SD) unless otherwise stated.

Total home environmental stimulation was assessed using Family Care Indicators (24 questions, potential range of scores: 0–24).

Fathers’ and mothers’ activities contributing to child development were scored based on five activities (yes=1/no=0): reading a book, telling a story, playing with toys, singing songs or rhymes, and drawing or counting with the child.

Maternal depressive symptoms were collected using the Self Reporting Questionnaire-20 (SRQ-20). A higher score indicates a more depressive condition.

Depressive symptoms presence was defined as score ≥ 7.

Quality of life (QoL) was measured using the WHO-BREF questionnaire. A higher score indicates better QoL.

Household violence against the mother was defined as any physical/psychological/verbal abuse by the husband or any family member of the household in the last one month.

There were no significant differences between mothers tested and those lost to follow-up regarding their background characteristics at the end of the intervention (supplementary table S1).

The mothers in the intervention group had lower depressive symptoms [adjusted regression coefficient, β; −1.53, Confidence interval, 95% CI −2.28, −0.80] compared to the mothers in the comparison group after one year of intervention. Household violence against mothers was found to be a predictor [β; 1.04 (95% CI; 0.29, 1.80)] of MDS in the adjusted linear regression model. Further analysis using structural equation modelling showed that violence against mothers was a mediator for MDS. Additionally, when we considered other covariates (mothers’ age, education, BMI, father’s education, children’s age and sex) in the model, both separately and together, we did not find any significant impact on MDS or their QoL (Table S2). The effect size was weak for MDS, whereas it was moderate for mothers’ QoL. Mothers in the intervention group had a higher QoL compared to those in the comparison group. Improvements were noted in physical health [β; 4.21 (95% CI; 1.71, 6.73)], psychological health [β; 3.14 (95% CI; 1.10, 5.19)], social relationships [β; 3.21 (95% CI; 0.76, 5.66)] and environment [β; 3.40 (95% CI; 1.37, 5.44)] compared to the no intervention group (Table II). Additionally, the findings were consistent with those from the regression model for each outcome using multiple imputation methods to address missing data (Table S3).

**Table II.** Intervention effect on maternal depressive symptoms and quality of life (*n* = 547).

Model	Outcomes	β coefficient	Confidence interval	P value	Effect size Cohen’s d
Effect of intervention on maternal depressive symptoms					
i	Maternal depressive symptoms	−1.53	−2.28, −0.80	<0.001	−0.20 (−0.03, −0.37)
Effect of intervention on maternal quality of life					
ii	Physical health	4.21	1.71, 6.73	0.002	0.30 (0.47, 0.13)
iii	Psychological health	3.14	1.10, 5.19	0.004	0.25 (0.42, 0.09)
iv	Social relationships	3.21	0.76, 5.66	0.013	0.21 (0.38, 0.05)
v	Environment	3.40	1.37, 5.44	0.002	0.28 (0.44, 0.11)

Linear regression was conducted for each outcome.

Respective baseline outcome, violence against mothers, maternal child development activities conducted with children, testers’ effect and clusters as random effect were adjusted for in each model.

Effect size was calculated using Cohen’s d, and it was calculated through mean difference divided by the standard deviation of the difference.

There was no correlation between baseline and post-intervention cognitive and language composite scores of the children and MDS at baseline. However, we found that baseline MDS were associated with both baseline and post-intervention household violence against mothers.

## Discussion

This study reported that a parenting intervention including nutrition education for child development, delivered through home visits, reduced MDS and improved all domains of mothers’ QoL in deprived urban settings in Bangladesh. We also found that household violence against mothers was a predictor of MDS.

In line with our findings, reduced MDS have been similarly observed in other parenting interventions aimed at improving children’s development in LMICs, including Bangladesh [18,19], Pakistan [20], Uganda [21], Jamaica [22] and India [23]. All these studies were different in many ways. Mehrin et al. delivered the parenting intervention through group sessions held fortnightly for one year by community clinic staff (rural government health system) in Bangladesh [18]. Tofail et al. integrated mental health and nutrition components with parenting interventions through group sessions at a household/community centre near the participants’ residences in urban slums of Bangladesh [19]. Yousafzai et al. combined nutrition and parenting interventions in Pakistan using the health system [20]. Singla et al. combined mental health components with a parenting intervention in Uganda [21]. Baker-Henningham et al. delivered parenting sessions weekly for one year at households with the help of community health aides and found that the intervention had a strong impact on MDS (effect size −0.43 SD) in Jamaica [22]. Andrew et al. conducted a parenting study in a poor urban population through weekly visits for 18 months in India [23]. On the other hand, a large number of parenting intervention studies did not report a reduction in MDS [28,29], as indicated by several systematic reviews and meta-analyses [30–32]. Thus, while reducing MDS should be an objective when designing a parenting programme, the context and various other factors must also be considered. These factors include the curriculum (parenting alone or in combination with other interventions), parental compliance, whether group or individual sessions, the frequency of sessions (weekly/bi-weekly), and the location where the sessions are conducted. Conducting further research, including systematic reviews and meta-analyses, is recommended involving subgroup analyses.

Although several parenting interventions have been shown to reduce MDS, the precise mechanisms of action remain unclear. Studies indicate that interventions designed specifically to boost the mother – infant relationship where post-natal depression is prevalent may benefit this relationship and potentially reduce the negative effects of maternal depression in the short term [51,52]. In our study, we found a 55% prevalence rate of MDS at enrolment (cut-off, SRQ ≥ 7 [38]). Culturally, it is assumed that childcare is one of the major responsibilities of mothers in LMICs, such as Bangladesh [53]. Interventions focused on children may change the family dynamics positively by encouraging parents to act appropriately, with support from other family members, for the sake of the children’s well-being [54,55]. Fathers also play an important role in contributing to their children’s development and in reducing MDS. The presence of both parents in the household is important, in terms of the child having a role model [56] and contributes to consistent parenting through increased monitoring, discipline [57] and time devoted to the child [58]. Interventions where both fathers and mothers are engaged may result in increased paternal engagement, which can enhance the care provided to both the child and the mother [59]. A father can care for his family through direct and indirect pathways. The direct pathway naturally refers to the father’s active involvement in positive interactions with his children, and indirect pathways typically denote intra-household processes in which the father affects the child indirectly, for example, by influencing the mother’s emotional and physical availability to the child [54,60]. Nonetheless, it is usually only mothers who attend parenting interventions, with fathers being less likely to engage in these programmes [61,62].

Extensive literature documents the association between violence against women and MDS [7]. Violence between spouses or partners has far-reaching effects on the family system as a whole, as children are witnesses to approximately 80–95% of such aggression [63,64]. In our previous article, we reported that the parenting intervention reduced household violence against mothers [33]. The findings of this study support that reducing violence against mothers may, in turn, contribute to a decrease in MDS. Previous literature supports the notion that a positive relationship between parents creates a conducive environment for children’s growth. Both mothers’ and fathers’ engagement in parenting is essential for achieving the best outcomes for children [65]. Researchers should focus on finding ways to reduce violence against mothers to foster a safe environment for the well-being of families and the healthy development of children, especially in LMICs where domestic violence is prevalent.

To our knowledge, this is the first study to report that parenting interventions can improve all domains of QoL of mothers. We were unable to identify any existing literature with comparable findings. This is an important finding for both mothers and children regarding their well-being [14]. Another study reported that the cash transfer programme alone can improve QoL [66]. Thus, the improvement of QoL among these disadvantaged mothers may be due to the synergy of cash transfers and improved parenting. This finding demonstrates the promise of this particular parenting programme in LMIC settings, especially in urban areas.

It is important to note some strengths and limitations of this study. A screening tool was used to measure depressive symptoms; thus, the findings cannot be generalised to patients with clinical depression. Data on violence against mothers and MDS were collected using recall methods during the preceding one month of the interview. Most other studies collected data on lifetime experience of violence. We tried to minimise recall bias in the violence data; however, these data may still be underreported due to issues such as the mothers’ reluctance to report their husbands’ abusive behaviour for fear of repercussions. Additionally, a one-month recall period for MDS does not allow for an assessment of depressive symptoms in mothers over time, as a recurrence of depressive episodes is highly likely [4]. Unconditional cash was provided for all mothers, making it was impossible to see the effect of unconditional cash alone on the outcomes of this study.

The study was designed following robust cluster randomisation methods, e.g. well-balanced groups (intervention and control), high adherence of participants to the intervention, and intention-to-treat analysis. Additionally, assessors were kept blind to the intervention, although the mothers might have alerted them during the assessment. One of the key strengths of the study is its external validity. The study was conducted in real-life settings, aiming to replicate practical service delivery in LMICs. These included the use of a government unconditional cash transfer programme, as well as interventions implemented by non-professional staff who are permanent residents of the study area and operate within community settings. These practical methods increase the external validity of the findings and their applicability to programme development.

This study is a step towards addressing the global gap in literature on evidence-based parenting interventions that may reduce MDS. Further research is essential.

Given these positive outcomes, policymakers should consider integrating parenting and nutrition education programmes into existing maternal and child health services. Additionally, scaling up such interventions nationally could play a crucial role in improving maternal mental health and overall well-being, ultimately contributing to better child development and public health outcomes. Further research is needed to explore the long-term impacts and cost-effectiveness of such interventions.

## Supplementary Material

supplementray_tables.docx
