# Supplementary material for: Parenting with nutrition education and unconditional cash reduce maternal depressive symptoms and improve quality of life: findings from a cluster randomised controlled trial in urban Bangladesh
Source: Glob Health Action. 2024 Nov 19;17(1):2426784. doi: 10.1080/16549716.2024.2426784 (PMC11578405; doi:10.1080/16549716.2024.2426784)
Supplement: supplementray_tables.docx [file ZGHA_A_2426784_SM8147.docx]

**Table (S1). Background information of tested and lost to follow-up children**

| **Background information** | **Tested** | **Lost to follow-up** |
| --- | --- | --- |
| Child stimulation environment score (Family Care Indicator score) | 5.78 (3.14) | 5.53 (3.30) |
| Father’s engagement with child development activities | 0.36 (0.77) | .39 (0.88) |
| Mother’s engagement with child development activities | 2.19 (1.43) | 1.94 (1.32) |
| Mother’s Body Mass Index | 22.75 (3.93) | 23.22 (4.51) |
| Mother’s Education | 7.27 (3.65) | 6.96 (3.38) |
| Mother’s depressive symptoms | 8.91 (4.87) | 8.43 (4.65) |
| Mother’s quality of life | 85.87 (10.10) | 85.78 (10.45) |

**Table S2. Multiple linear regression model to see the effect of the intervention (considering all covariates)**

| Covariates | B-Coefficient | 95% Confidence Interval |
| --- | --- | --- |
| Intervention | -1.50 | -2.24, -0.74 |
| Mother’s information at enrolment | | |
| SRQ score | 0.44 | 0.37, 0.51 |
| Violence against mothers | 1.02 | 0.29, 1.76 |
| BMI | 0.01 | -0.05, 0.07 |
| Education | -0.07 | -0.18, 0.04 |
| Child development activity | 0.37 | -0.71, 1.45 |
| Father’s education | -0.06 | -0.18, 0.05 |
| Child’s age | -0.02 | -0.11, 0.08 |
| Child’s sex (girls) | 0.49 | -0.32, 1.30 |
| Adjusted R^2^ | 0.38 | |
| SRQ; Self-Reported Questionnaire BMI; Body Mass Index | | |

**Table S3. Intervention effect on maternal depressive symptoms and quality of life (n=587) from multiple imputation method**

| Model | Outcomes | β  coefficient | Confidence interval | P  value |
| --- | --- | --- | --- | --- |
| i | Maternal depressive symptoms | -1.57 | -2.27, -0.87 | <0.001 |
| ii | Physical health | 4.05 | 1.91, 6.19 | <0.001 |
| iii | Psychological health | 3.11 | 1.23, 4.98 | 0.001 |
| iv | Social relationships | 3.16 | 0.85, 5.48 | 0.007 |
| v | Environment | 3.51 | 1.72, 5.29 | <0.001 |
| *To address missing data, we employed multiple imputation by chained equations (MICE), generating 10 imputed datasets. After imputation, we used maximum likelihood (ML) estimation to fit a mixed-effects model to each of the imputed datasets. Following model estimation, the results from the 10 imputed datasets were pooled using Rubin’s rules to combine the parameter estimates and standard errors, ensuring valid statistical inference accounting for the uncertainty due to missing data. This pooled analysis allowed for unbiased estimates of both fixed and random effects.  *Respective baseline outcome, violence against mothers, maternal child development activities conducted with children, testers’ effect and clusters as random effect were adjusted for in each model. | | | | |
